# Cognitive Stimulation for Individuals with Parkinson's Disease Dementia Living in Long-Term Care: Preliminary Data from a Randomized Crossover Pilot Study

**DOI:** 10.1155/2018/8104673

**Published:** 2018-12-02

**Authors:** Ann-Kristin Folkerts, Miriam E. Dorn, Mandy Roheger, Marco Maassen, Janneke Koerts, Oliver Tucha, Mareike Altgassen, Alexander T. Sack, Diede Smit, Lena Haarmann, Elke Kalbe

**Affiliations:** ^1^Medical Psychology | Neuropsychology and Gender Studies & Center for Neuropsychological Diagnostics and Intervention (CeNDI), University Hospital Cologne, Kerpenerstraβe 62, 50937 Cologne, Germany; ^2^Verpleeghuis Lückerheide, Residential Group for People with Parkinson or Cognitive Impairment and Dementia, St. Pieterstraat 145, 6463 CS Kerkrade, Netherlands; ^3^Department of Clinical and Developmental Neuropsychology, Faculty of Behavioural and Social Sciences, University of Groningen, Grote Kruisstraat 2/1, 9712 TS Groningen, Netherlands; ^4^Donders Institute for Brain, Cognition and Behavior, Radboud University, Comeniuslaan 4, 6525 HP Nijmegen, Netherlands; ^5^Faculty of Psychology and Neuroscience & Maastricht Brain Imaging Centre, Maastricht University, Minderbroedersberg 4-6, 6211 LK Maastricht, Netherlands

## Abstract

**Background:**

While the efficacy of cognitive stimulation (CS) has been demonstrated in patients with dementia, no study has included patients with Parkinson's disease dementia (PDD).

**Objective:**

For the first time, this randomized crossover pilot study examined the feasibility and potential effects of CS in PDD.

**Methods:**

All residents of a PDD-specific long-term care unit in the Netherlands that were eligible for the study (*n*=12) were randomly allocated to group A (*n*=6) receiving CS (eight weeks, twice weekly for 60 minutes) or group B (*n*=6) receiving usual care (control group, CG). The CG participated in CS afterwards, resulting in an experimental group (EG), consisting of *n*=12. Pre- and postassessments and a six-week follow-up (FU) were conducted for cognition, neuropsychiatric symptoms, quality of life (QoL), and activities of daily living (ADL) outcomes.

**Results:**

Between-group analysis with difference scores from pre- to posttest revealed a group difference for global cognition (CERAD total score) favoring the EG, with a moderate effect size and a *p* value just failing to reach statistical significance (*p*=0.067; *r* = 0.43). A further statistical trend was observed for neuropsychiatric symptoms, again with a moderate effect size (*p*=0.075; *r* = 0.42). Within-group analyses indicated improvement only in the EG with large effects also just failing to reach significance for global cognition (short term, *p*=0.060; *r* = 0.70) as well as for depression (long term, *p*=0.072; *r* = 0.61). ADL deteriorated significantly at FU in the EG (*p*=0.014; *r* = 0.71).

**Conclusions:**

Although our data are preliminary due to the small sample size, this study shows that CS is feasible and potentially effective for cognitive and noncognitive outcomes in PDD patients. Randomized controlled trials with larger sample sizes are needed to confirm these promising results.

## 1. Introduction

Parkinson's disease dementia (PDD) is highly prevalent [1] and impacts patients, caregivers, and the health-care system [2]. Currently, there are limited pharmacological treatments available for PDD [3, 4]. Thus, nonpharmacological interventions to treat or prevent cognitive symptoms are increasingly identified as promising therapy options in Parkinson's disease (PD) [5, 6].

Meta-analyses of controlled and randomized controlled trials (RCTs) have shown the efficacy of cognitive training on cognitive and noncognitive symptoms in nondemented PD patients [5, 7]. Similarly, one pilot study on goal-oriented cognitive rehabilitation in PDD patients revealed benefits in, e.g., self-rated goal attainment, mood, and quality of life (QoL) [8]. Surprisingly, while cognitive stimulation (CS) (i.e., cognitively stimulating activities in small groups targeting the stabilization or improvement of global cognitive and social functioning primarily in patients with dementia [9, 10]) has been demonstrated to be effective in non-PD dementia patients [10–12], to the best knowledge of the authors, no study has examined its effects in PDD patients (although a study protocol has been recently published [13]).

Thus, we conducted a pilot randomized crossover trial of CS in PDD patients to examine the feasibility and potential effects. We hypothesized that CS is feasible in this context and that the PDD patients participating in a CS program would show benefits in cognition, neuropsychiatric symptoms, and QoL.

## 2. Materials and Methods

The reporting of this pilot RCT follows the CONSORT guidelines [14]. The study was registered in the German Clinical Trials Register (DRKS; ID: DRKS00011776) and was approved by the Ethics Committee of Lückerheide initiated by the MeanderGroep, Kerkrade, the Netherlands. Research was conducted in accordance with the 1975 Declaration of Helsinki. Before starting with data assessment, legal representatives gave informed written consent. No published study protocol exists.

### 2.1. Setting and Participants

The data assessment occurred between January 2017 and June 2017 in a long-term care facility in Kerkrade, the Netherlands. This institution is unique as, to the best knowledge of the authors, it is the only facility with a specific care unit for residents with PD and cognitive dysfunctions. At the beginning of the study, 21 PD patients lived in the unit. The aim was to include as many patients as possible from the care unit. Therefore, the inclusion criteria were defined rather broadly as (i) being a resident in the PD care unit, (ii) idiopathic PD diagnosed by a neurologist or psychiatrist, (iii) cognitive dysfunction operationalized by the Mini-Mental State Examination (MMSE; 10 to 25 points) [15], (iv) Dutch as the native language or very good Dutch language skills, (v) only slightly restricted or corrected-to-normal vision and hearing, and (vi) the provision of written consent from a legal representative to participate in the study.

The exclusion criteria were (i) depressive symptoms, which were not compatible with the participation in a CS program, (ii) bedridden residents, as this is an obstacle for participating in the group intervention, (iii) life-threatening illness, (iv) history of alcohol or drug abuse in the last three years, and (v) acute suicidal tendencies or acute psychotic symptoms.

### 2.2. Study Design

**Figure 1:**
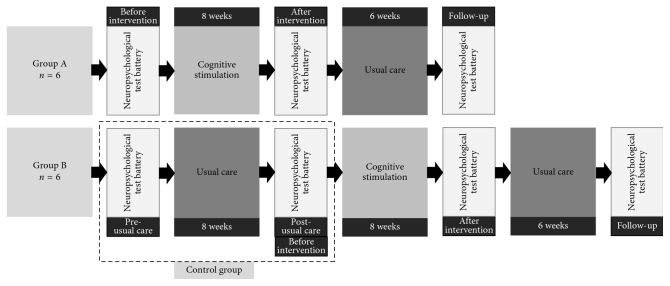
Study design.

The study was designed as a randomized crossover trial with two groups (Figure 1). Group A started with participation in the CS program for eight weeks, while group B followed the usual care. After eight weeks, the condition for both groups changed. For group A, assessments took place before and after the intervention (preintervention vs. postintervention) as well as six weeks after the intervention had finished (follow up, FU). During the FU period, group A received the usual care. For group B, assessments took place before and after eight weeks of usual care (pre- vs. post-usual care/preintervention), as well as after the intervention (postintervention), and six weeks thereafter (follow up, FU). The group A phase between postintervention and FU was not added to the control group (CG), as no or limited washout effects could be expected for our nonpharmacological intervention. Assessments were scheduled one week before and after the intervention/waiting period, respectively. All assessments and intervention sessions were conducted with patients in the ON state.

The allocation to group A or B was conducted by an independent member of the research group who was not involved in the study. Each group consisted of six patients. After inclusion, participants were matched according to their MMSE total score. The pairs of matched individuals were randomly allocated to either group A or B by picking a note with the patient's identification code, which was composed of two random letters and two random numbers, from an urn. The person who was picked first was assigned to group A and the second one to group B. Each group was split into groups of three to meet the group size requirement for the CS program.

The participants and facilitators for the CS program were not blinded. Both outcome assessors were blinded at the beginning of the data collection, but at the subsequent time points of assessment, only one assessor was blinded to the group allocation for organizational reasons. Nursing staff involved in external assessments were not blind to the group allocations.

### 2.3. Intervention Program

The intervention comprises a modification of the structured CS program “NEUROvitalis senseful” [16], which was used in a study with patients with dementia living in a nursing home [17], and is a further development of the standardized cognitive training program “NEUROvitalis” [18]. NEUROvitalis senseful is offered to small groups (3 to 5 persons) over a period of eight weeks, twice per week for 60 minutes. For PDD patients' suitability, the exercises were adapted according to the typical cognitive and psychomotor profile of PD patients. Thus, we added more cognitive exercises targeting executive and visual-spatial functions, as well as fine motor skills training (Supplementary Table 1). For this study, the manual with detailed description for every session was translated to Dutch by native speakers with excellent knowledge of the German language and expertise in clinical neuropsychology. The program was carried out in the PD care unit by Dutch psychologists with a Bachelor's degree who were trained by one of the developers of the program (AKF).

### 2.4. Control Group (CG)

The CG received the usual PD unit care including a variety of nonpharmacological interventions such as sports, music, and arts. These activities were open and voluntary for all residents throughout the study.

### 2.5. Outcomes

Cognitive functioning was assessed with the Dutch version of the CERAD test battery [19] (plus a word fluency test [20] and the Trail Making Test (TMT) [21], which are included in the German version “CERAD Plus” [22]). The CERAD total score as an index for global cognition (max. 111 points) was calculated according to Seo et al. [23]. Additionally, we chose the Clock Drawing Test [24] to test visuoconstructive and executive functions.

Neuropsychiatric symptoms were assessed with the Neuropsychiatric Inventory (NPI) [25], and depression was assessed with the Geriatric Depression Scale (GDS) [26] and the Cornell Scale for Depression in Dementia (CSDD) [27]. ADLs were measured with the Barthel Index [28] and QoL with the Dutch version of the EQ-5D-5L [29] as self- and external ratings and the 37-item version of the QUALIDEM [30] as a second external assessment. The QUALIDEM total score was calculated [31]. For a detailed description of the (neuro‐)psychological test battery, see Supplementary Table 2.

The neuropsychological examinations were administered in a fixed order by two Dutch psychologists with a Master's degree trained in neuropsychological testing. The external assessments were conducted as interviews with the nursing staff most involved in the patients' care.

Further data, including demographics and clinical details, were collected from the residents' files. The levodopa equivalent daily dose (LEDD) was calculated with the Levodopa Equivalent Dose Calculator (http://www.parkinsonsmeasurement.org/toolBox/levodopaEquivalentDose.htm). The Charlson Comorbidity Index (CCI) was calculated to predict the risk for death from comorbid diseases [32]. PDD was diagnosed according to the Movement Disorders Society (MDS) task force Level I guidelines [33].

### 2.6. Statistical Analysis

SPSS 25 statistics software (IBM) was used for data analyses. Only patients who completed at least 11 sessions of the CS program were included in analyses. No further techniques for dealing with missing data (e.g., imputations) were used. As the number of participants in the special setting was naturally limited and this was a pilot study, no a priori sample size calculation was performed. A post hoc power analysis was calculated using G∗power 3.1 [34]. Analyses were performed for the main outcomes of this study: global cognition, neuropsychiatric symptoms, depression, ADLs, and quality of life.

The alpha level was set at 0.05 for all analyses. An adjustment of *p* values (0.05) using Bonferroni correction within those outcomes which had several subscores (i.e., depression and QoL) was applied. The effect size *r* is reported indicating small effects from *r* ≥ 0.1 to *r* < 0.3, medium effects from *r* ≥ 0.3 to *r* < 0.5, and large effects from *r* ≥ 0.5 [35]. Mean scores and standard deviations for the baseline characteristics of groups A and B were shown. We used *t*-tests for normally distributed data and the Mann–Whitney *U* test for nonnormally distributed data, respectively. Gender and Hoehn and Yahr (H&Y) stages are shown as frequencies and compared using the chi-square statistics.

Due to the specific crossover design, the total number of participants in the CS group increases (group A + group B, *n*=12). The Mann–Whitney *U* test was used to compare between-group CS effects. For this analysis, the score differences from postintervention to preintervention and from post-usual care to pre-usual care were calculated, respectively. The Wilcoxon signed-rank test was used for within-group differences from post- to preintervention (short-term effects) and from FU to preintervention (long-term effects) for CS, as well as from post-usual care to pre-usual care (short-term effects) for the CG. Due to the crossover design, no long-term effects analysis could be performed for the CG, as there was no FU in that group.

## 3. Results

**Figure 2:**
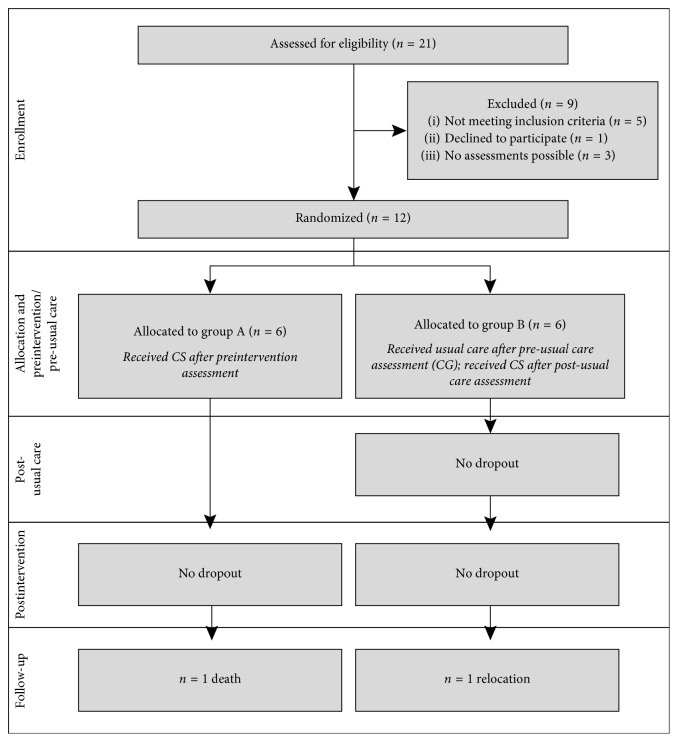
Flowchart of the participants recruited for this study.

Figure 2 illustrates the sample size in this study. All 21 residents of the PD care unit were screened for eligibility, 12 of which could be included in the study. Two subjects (one person per group) dropped out at the FU assessment (dropout rate: 16.7%). The residents attended between 11 and 16 CS sessions (participation score: 92.7%). The nursing staff was very interested in the study process and supported its organization. The impression of the trainer was that the residents were also pleased to participate, and several individuals mentioned that they enjoyed the CS sessions. During the study process, all subjects (except for one resident) participated in activities provided by the institution such as arts or music (Supplementary Table 3).

At baseline, all patients were classified as having PDD according to the MDS criteria [33], except for the criteria of absence of major depression (GDS ≥ 5 points), which was reached in 10 of 12 patients. Groups A and B were comparable with regard to all baseline characteristics (Table 1).

No adverse effects occurred in relation to CS participation. All outcomes for both groups with between- and within-group statistics are presented in Table 2 (data were largely complete).

### 3.1. Between-Group Effects

The between-group analysis revealed a group difference favoring CS which just failed to reach statistical significance for the CERAD total score (*p*=0.067; *r* = 0.43) with a moderate effect size, and also a statistical trend for the NPI total score (*p*=0.075; *r* = 0.42), again with a moderate effect size. The post hoc power analysis for the between-group effects demonstrated a statistical power of 6.6% for small effects, 21.1% for moderate effects, and 56.1% for large effects.

### 3.2. Within-Group Effects

The within-group analysis showed a positive trend, which just failed to reach statistical significance but showed a strong effect size for the CERAD total score (*p*=0.060; *r* = 0.70) demonstrating an improvement of the CS group (*n*=12) from pre- to postintervention. Preintervention to FU score comparisons revealed an improvement for the GDS (*p*=0.072; *r* = 0.61), which again just failed to reach statistical significance and showed a large effect size. A significant deterioration was shown for the Barthel index (*p*=0.014; *r* = 0.71).

The post hoc power analysis for the within-group CS effects demonstrated a statistical power of 9.5% for small effects, 48.9% for moderate effects, and 94.2% for large effects.

**Figure 3:**
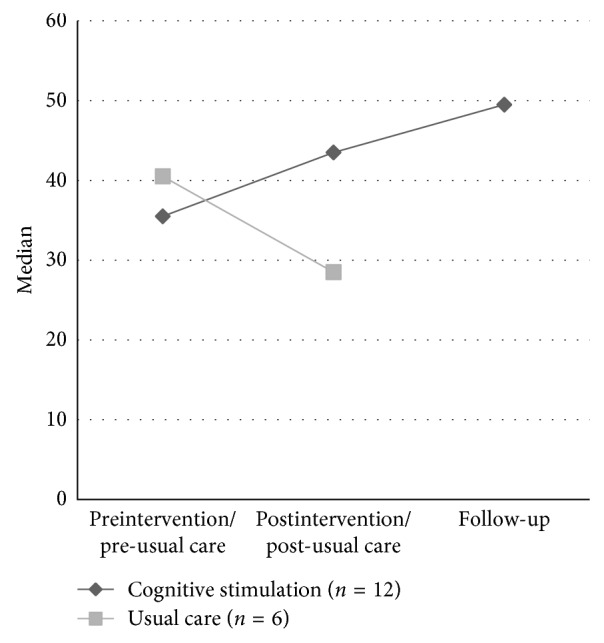
Median change of the CERAD total score (max. 111 points; higher scores indicating better performance) of the experimental (EG) and control group (CG) from preintervention/pre-usual care to postintervention/post-usual care to follow-up assessment.

**Figure 4:**
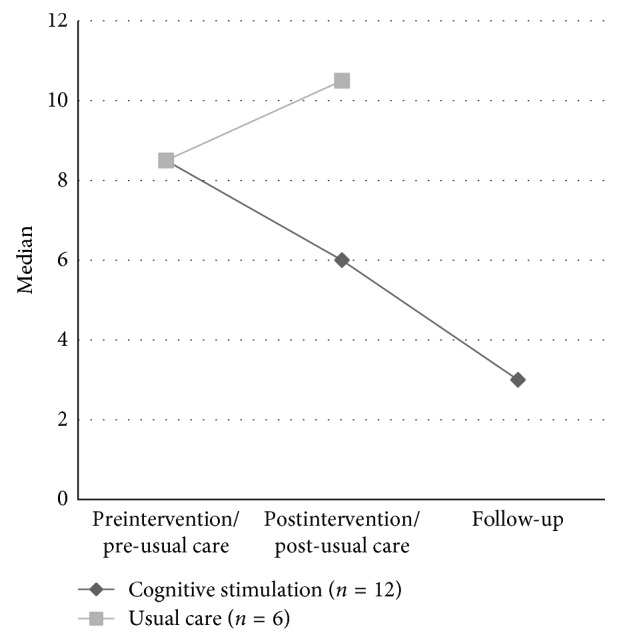
Median change of the Geriatric Depression Scale (GDS; max. 15 points; lower scores indicating less depressive symptoms) of the experimental (EG) and control group (CG) from preintervention/pre-usual care to postintervention/post-usual care to follow-up assessment.

The comparison of the pre- and post-usual care assessment of the CG revealed no significant results. Figures 3 and 4 illustrate the changes in the CERAD total score and the GDS for the CS and the CG for all assessment points. The descriptive data of the cognitive subtests and the QUALIDEM subscores are presented in Supplementary Table 4.

## 4. Discussion

This randomized crossover pilot study examined short- and long-term effects of an eight-week CS program for long-term care residents with PDD.

Although our data must be regarded as preliminary due to the small sample size, it shows that CS for PDD patients might be an effective therapy option. First of all, our findings indicate that CS is a safe therapy option for patients with PDD and has possible positive short-term effects on cognition and neuropsychiatric symptoms for residents with PDD in long-term care. These benefits were demonstrated with between- and within-group analyses, with moderate to strong effect sizes for all effects. Furthermore, preliminary long-term CS benefits were found at the six-week FU for depressive symptoms, again with a strong effect size. Finally, a significant deterioration was observed from baseline to FU in ADL performance of the CS group.

Despite the small sample size, this pilot trial was able to identify potential beneficial effects on cognitive and noncognitive parameters, which are consistent with CS meta-analyses in non-PD dementia patients showing positive effects on global cognition [10–12]. Additionally, we revealed a decrease in neuropsychiatric symptoms and depression, corroborating the results of Folkerts et al. [12] who found CS benefits for patients with dementia living in long-term care. Nevertheless, another meta-analysis did not find such an effect [10]. However, a positive effect on QoL, which was demonstrated, e.g., in the Cochrane review by Woods et al. [10], could not be revealed in this study. Future studies will have to elucidate whether CS has the potential to improve the QoL in PDD. One possible explanation for the lack of QoL benefits could be that QoL questionnaires were used that were not sensitive for this specific patient group. Thus, the development and use of specially designed QoL questionnaires for PDD patients is an urgent purpose for future studies. Also, further trials with larger PDD patient sample sizes are needed to confirm the potential of CS in this group.

Notably, our data show a significant deterioration of ADL in the CS group from preintervention to FU. As PDD is a progressive neurodegenerative disease, and the impact on daily functioning is its key characteristic, this decline can most probably be ascribed to the progress of the disease. However, in the light of this decline, the benefits of CS appear to be even more remarkable. Unfortunately, as an FU assessment for the CG is lacking, no between-group analysis was possible. Therefore, potentially different rates of decline in ADL will need to be the subject of future research.

### 4.1. Feasibility

The intervention integrated well into the daily routine of this specialized PD care unit, despite the fact that the residents' weekly schedule included further activities. This is verified by the high participation score ≥ 94%. We, therefore, conclude that the CS program is feasible for PDD patients. Furthermore, participants reported that they enjoyed the CS sessions. The nursing team was interested in the study, helped with organizing the assessments, provided information about each patient within the external assessments, and arranged the CS sessions. The CS materials remained in the institution after the final assessment, and the program is still provided to the residents.

### 4.2. Limitations and Strengths of the Study

Some limitations need to be considered when interpreting our findings. First, the power of our study is limited due to the small sample size. As we used the unique possibility to study CS effects in a specialized care unit for PDD patients, no a priori sample size estimation was possible. However, it should be noted that our post hoc power analyses indicated that the power for the within-group effects of the CS group was satisfactory at 94% for large effects. Furthermore, it seems remarkable that despite the small sample size, significant clear trends with moderate to large effect sizes were found.

The crossover design, which was used to enlarge our sample size, may be associated with some critical aspects, especially in a study of the effects of a nonpharmacological intervention, where no or limited washout effects could be expected. For example, no CG was available for analyzing between-group differences of the long-term effects. Furthermore, the patients had different numbers of measurement time points (three vs. four measurements). As PDD is a progressive disease, a possible deterioration in group B from pre-usual care to post-usual care assessment needs to be considered. Finally, one of the two assessors was not blinded to group allocation. Full blinding should be achieved in future studies.

As indicated, participants reported that they enjoyed participation in the CS program. However, a limitation is that, we did not systematically collect data on the important patient-related outcome. Therefore, future studies should include measures of the individuals' motivation and fun while participating in a CS program. Possible approaches may include the use of training diaries and specific questionnaires as well as short qualitative interviews at the end of the CS program involving both participants and relatives/nursing staff for self- and external ratings.

A particular strength of the study is that it is the first to show preliminary data for the beneficial effects of CS in PDD patients. The consideration of the CONSORT guidelines for study reporting is a further strength. Finally, we provide FU data to investigate possible long-term effects, which has been lacking in many CS studies [10].

## 5. Conclusions

In conclusion, CS seems to be a promising and safe nonpharmacological intervention to enhance cognitive and noncognitive symptoms in PDD patients in long-term care. Further research with larger sample sizes and longer FU periods are of high importance to confirm and specify the effects of CS for out- and inpatients with PDD. The best-suited frequency and duration of CS sessions should be identified. Predictor analyses could help to identify factors influencing CS benefits. Finally, health economic evaluations will be necessary.

## Figures and Tables

**Table 1:** Baseline sociodemographic and clinical characteristics of the study sample.

	Group A (*n*=6)	Group B (*n*=6)	*p* value
Age (years)	76.67 ± 5.58	76.50 ± 8.94	0.970
Gender (male/female)	5/1	5/1	1.000
Years of education	10.17 ± 1.60	9.50 ± 0.55	0.445
Family status			0.392
** **Unmarried	0	1	
** **Married	3	1	
** **Divorced	0	1	
** **Widowed	3	3	
Inpatient (months)	16.50 ± 14.95	10.33 ± 5.50	0.394
Hoehn & Yahr stage			0.255
** **1–1.5	0	0	
** **2–2.5	2	1	
** **3	0	2	
** **4	2	2	
** **5	2	1	
Months since PD diagnosis	72.00 ± 37.18	74.00 ± 48.84	0.938
Months since dementia diagnosis	26.17 ± 28.20	27.67 ± 12.66	0.908
LEDD	290.17 ± 236.60	239.67 ± 228.06	0.714
CCI	3.50 ± 3.78	2.33 ± 1.03	0.937
Number of antidementiva	0.83 ± 0.98	1.00 ± 0.63	0.699
Number of antidepressiva	0.83 ± 1.17	0.42 ± 0.66	0.699
Number of other medications	11.11 ± 3.27	10.42 ± 2.33	0.699
MMSE (max. 30 points)	17.50 ± 5.75	18.17 ± 5.35	0.839
CERAD total score (max. 111 points)	39.17 ± 9.37	38.50 ± 13.78	0.924
GDS (max. 15 points)	6.00 ± 2.00	8.67 ± 4.46	0.394

Values are presented as the mean ± standard deviation or frequency. Abbreviations: LEDD, levodopa equivalent daily dose; CCI, Charlson Comorbidity Index; MMSE, Mini-Mental State Examination; CERAD, Consortium to Establish a Registry for Alzheimer's Disease; GDS, Geriatric Depression Scale.

**Table 2:** Medians and ranges of all main outcomes for all time points of measurement differentiated for intervention and control group and related within- and between-comparisons.

	Cognitive stimulation (*n*=12)							Usual care (*n*=6)				Cognitive stimulation vs. usual care	
	Preintervention	Postintervention	Within-comparisons		Follow-up	Within-comparisons		Pre-usual care	Post-usual care	Within-comparisons		Between-comparisons	
	Median (range)	Median (range)	*p*	*r*	Median (range)	*p*	*r*	Median (range)	Median (range)	*p*	*r*	*p*	*r*
*Cognition*													
CERAD total score^a^	35.50 (20–61)	43.50 (23–73)	*0.060*	0.70	49.50 (15.00–69.00)^2^	0.161	0.40	40.50 (19–58)	28.50 (20–57)	0.345	0.27	*0.067*	0.43

*Depression*													
GDS^b^	8.50 (3–14)	6.00 (3–12)^5^	0.950	0.21	3.00 (1–11)^3^	*0.072*	0.61	8.50 (4–14)	10.50 (3–14)	0.832	0.33	0.808	0.22
CSDD^b^	3.00 (0–18)	2.00 (0–15)	1.000	0.04	1.00 (0–14)^4^	0.320	0.41	5.00 (4–8)	4.00 (2–13)	1.000	0.04	0.500	0.28

*Neuropsychiatric symptoms*													
NPI total score (items A–L)^b^	10.00 (1–35)^5^	8.00 (0–51)^5^	0.439	0.22	7.50 (0–41)^4^	0.575	0.16	10.00 (1–21)^1^	13.00 (5–35)	0.414	0.33	*0.075*	0.42
*Activities of daily living (ADL)*													
Barthel Index^a^	14.50 (2–20)	15.00 (4–20)^5^	0.572	0.16	13.50 (0–18)^4^	*0.014*	0.71	15.50 (9–20)	15.50 (9–20)	0.414	0.33	0.961	0.02

*Quality of Life*													
EQ-5D-5L index value^a^	0.68 (0.09–1.00)	0.71 (0.09–1.00)^5^	1.000	0.06	0.74 (0.05–0.83)^4^	1.000	0.34	0.71 (0.11–0.99)	0.71 (0.16–1.00)	0.545	0.65	1.000	0.08
EQ-5D-5L-VAS^a^	55.00 (5–85)	65.00 (45–80)^5^	0.700	0.43	55.00 (35–90)^3^	1.000	0.28	50.00 (40–80)	55.00 (5–75)	1.000	0.04	1.000	0.24
EQ-5D-5L index value (proxy)^a^	0.73 (0.02–1.00)	0.73 (0.14–0.93)	1.000	0.15	0.76 (0.09–1.00)^3^	1.000	0.20	0.72 (0.51–0.91)	0.73 (0.54–1.00)	1.000	0.17	1.000	0.12
EQ-5D-5L-VAS (proxy)^a^	57.50 (10–90)	50.00 (25–80)	1.000	0.19	60.00 (40–80)^3^	1.000	0.27	50.00 (35–60)	52.50 (10–70)	1.000	0.17	1.000	0.06
QUALIDEM total score^a^	16.77 (8.53–22.25)^4^	17.24 (13.36–24.83)^5^	0.430	0.50	17.43 (7.70–23.50)^3^	0.455	0.49	17.96 (13.79–22.52)^1^	16.32 (13.45–17.78)^1^	1.000	0.30	0.995	0.33

Statistical trends are in italics. Abbreviations: CERAD, Consortium to Establish a Registry for Alzheimer's Disease; GDS, Geriatric Depression Scale; CSDD, Cornell Scale for Depression in Dementia; NPI, Neuropsychiatric Inventory; VAS, visual analog scale. ^a^Higher scores indicate a better performance. ^b^Lower scores indicate a better performance. ^1^ *n*=5; ^2^ *n*=8; ^3^ *n*=9; ^4^ *n*=10; ^5^ *n*=11.

## Data Availability

The data used to support the findings of this study are available from the corresponding author upon request.

## Supplementary Materials

Supplementary Materials Supplementary Table 1: structure and elements of the cognitive stimulation program NEUROvitalis senseful; Supplementary Table 2: overview of the neuropsychological test battery; Supplementary Table 3: activities of the residents: a typical week as reported by the nursing staff; Supplementary Table 4: medians and ranges of all cognitive subtests and QUALIDEM subscores for all time points of measurement differentiated for CS group and CG.
