# Supplementary material for: Cognitive Stimulation for Individuals with Parkinson's Disease Dementia Living in Long-Term Care: Preliminary Data from a Randomized Crossover Pilot Study
Source: Parkinsons Dis. 2018 Dec 2;2018:8104673. doi: 10.1155/2018/8104673 (PMC6304852; doi:10.1155/2018/8104673)
Supplement: Supplementary Materials — Supplementary Table 1: structure and elements of the cognitive stimulation program NEUROvitalis senseful; Supplementary Table 2: overview of the neuropsychological test battery; Supplementary Table 3: activities of the residents: a typical week as reported by the nursing staff; Supplementary Table 4: medians and ranges of all cognitive subtests and QUALIDEM subscores for all time points of measurement differentiated for CS group and CG. [file 8104673.f1.pdf]

Supplementary Material

Cognitive stimulation for individuals with Parkinson’s disease dementia living in long-term care: Preliminary data from a randomized crossover pilot study

Folkerts, Dorn et al.

Supplementary Table 1

Structure and elements of the cognitive stimulation program NEUROvitalis senseful.

No.	Welcome & mood scale (5 min.)	Exercises of cognitive functions (25 min.)	Executive functions	Memory	Space cognition	Social cognition	Fine motor skills training (10 min.)	Sensory stimulation (15 min.)	Auditory perception	Tactile perception	Olfactory perception	Visual perception	Mood scale & goodbye (5 min.)
1		Think differently I	X				Double dutch	Describe and name sounds + assignment to categories	X				
2		Category memory game I		X			Knot game	Describe and name tactile material + assignment to categories		X			
3		Think differently II	X				Proverbs jigsaw puzzles	Describe and name fragrances + assignment to categories			X		
4		Category memory game II		X			Fishing game	Fruit basket and box of vegetables – Version 1				X	
5		City map game I			X		Double dutch	Describe and name sounds	X				
6		Category memory game III		X			Knot game	Describe and name tactile material		X			
7		Think differently III	X				Proverbs jigsaw puzzles	Describe and name fragrances			X		
8		City map game II			X		Fishing game	Fruit basket and box of vegetables – Version 2				X	
9		Everyday situations				X	Double dutch	Describe, name and evaluate sounds	X				
10		Sorting cards by colour, shape and number I	X				Knot game	Describe, name and evaluate tactile material		X			
11		City map game III			X		Proverbs jigsaw puzzles	Describe, name and evaluate fragrances			X		
12		Basic emotions				X	Fishing game	Fruit basket and box of vegetables – Version 1				X	
13		Sorting cards by colour, shape and number II	X				Double dutch	Describe and name sounds + assignment to pictures	X				
14		Basic emotions & everyday situations				X	Knot game	Describe and name tactile material + assignment to pictures		X			
15		Meaningful pictures		X			Proverbs jigsaw puzzles	Describe and name fragrances + assignment to pictures			X		
16		Sorting cards by colour, shape and number III	X				Fishing game	Fruit basket and box of vegetables – Version 2				X	

Supplementary Table 2

Overview of the (neuro)psychological test battery.

Assessment/ questionnaire	Description
**CERAD Plus [1,2,3,4]**	The CERAD Plus contains 11 subtests (semantic verbal fluency: animals in one minute, modified Boston Naming Test, MMSE, word list learning, recall, and recognition, constructional praxis, delayed constructional praxis, Trail Making Test, and letter verbal fluency: P-words in one minute) targeting global cognition, attention, executive functions, verbal and non-verbal memory, visuoconstructive abilities, and language with higher scores indicating better performance. The CERAD total score (maximum 111 points) was calculated as a value for global cognition according to Seo et al. [5]. The total score does not include the "Plus" tests, but comprises the subtests verbal fluency, Boston Naming Test, word list learning, recall, and recognition, as well as constructional praxis and recall.
**Clock Drawing Test [6,7]**	The Clock Drawing Test is an assessment of visuoconstructive and executive functions. The patients receive a piece of paper with a large circle on it and were asked to put all the numbers in. Subsequently, patients had to draw in the clock hands and indicate a time of 11:10. The scoring of the drawn clock is evaluated from 1 to 10 points, where a higher score is related to better performance.
**Geriatric Depression Scale (GDS) [9]**	The Geriatric Depression Scale consists of 15 mood related items with higher scores (maximum 15 points; 5 points are defined as the cutoff for mild depression) indicating more depressive symptoms.
**Cornell Scale for Depression in Dementia (CSDD) [10]**	The questionnaire, which is used as an external assessment, contains 19 items covering mood-related signs, behavioral disturbances, physical signs, cycling functions, and ideational disturbances. Each item can be scored as "a = unable to evaluate, 0 = absent, 1 = mild or intermittent, or 2 = severe". Higher scores (maximum 38 points) describe more depressive symptoms.
**Neuropsychiatric Inventory (NPI) [8]**	The Neuropsychiatric Inventory (NPI) comprises 12 items for delusions, hallucinations, agitation, dysphoria, anxiety, apathy, irritability, euphoria, disinhibition, aberrant motor behavior, nighttime behavior disturbances, and appetite and eating abnormalities. For each item, the frequency, severity, domain score (frequency x severity), and perceived burden were rated. A total score considering all 12 items was calculated.
**Barthel Index [11,12]**	The Barthel Index is used as an external assessment. Ten of the most prominent ADLs were queried and evaluated according to the level of independence: feeding, transfer, personal toilet, getting on and off toilet, bathing, walking, using stairs, dressing, as well as controlling bowels and bladder. Higher scores (maximum 20 points) are related to better ADL performance.
**EQ-5D-5L [13]**	The EQ-5D-5L consists of two tasks. The descriptive system is comprised of five dimensions (mobility, self-care, usual activities, pain/discomfort, and anxiety/depression) that are valued at five levels: no (1)/slight (2)/moderate (3)/severe (4)/extreme (5) problems. This five-digit number describes the patient's health related quality of life whereas the number 11111 indicates no problems in any of the five dimensions, 55555 indicates severe problems in all of these areas. The second task involves a 20 cm vertical visual analog scale with the endpoints "the best health you can imagine" and "the worst health you can imagine". The subject is asked to mark an X on the scale to describe today's health status.
**QUALIDEM [14,15]**	The QUALIDEM consists of 40 items. Each item can be assigned to one of nine sub-scores: care relationship, positive affect, negative affect, restless tense behavior, positive self-image, social relations, social isolation, feeling at home, and have something to do. The rater must decide whether the item occurs never (0), rarely (1), sometimes (2) or frequently (3). The total score calculation was suggested by Verbeek and colleagues [16] with higher scores (maximum 27 points) being related to a better quality of life (QoL).

Supplementary Table 3

Activities of the residents: a typical week as reported by the nursing staff.

	Patient #	Cognitive intervention	Physical exercise	Music (e.g. singing, orchestra)	Arts (e.g. painting, crafting)	Walking	Social activities (e.g. visits by relatives)	Reading the newspaper	Further activities (e.g. shopping, manicure, lottery)
Group A	1	1	4	0.5[1]	0	1	0	0	0
	2	0	0	1	0	0	0	0	0
	3	0	0	0	0	0.25[2]	7	0	0
	4	0	0	0	0	4	0	7	0
	5	1	2	0	X	0	1	0	0
	6	0	0	0	0	1	2	0	1
Group B	1	0	2	1	0	2	0	0	1
	2	0	0	2	0	0	0	0	0
	3	0	0	0	0	0	0	0	0
	4	0	2	0	0	1	0	0	0
	5	0	0	0	0	5	0	0	1
	6	0	0	0	1	0	0	0	1
Total No.		2	10	4,5	1+	14.25	10	7	4

Numbers in cells indicate the number of weekly attended sessions in each activity; X indicates that the number of weekly attended sessions was not documented.

[1]Patient attended activity once in two weeks; [2]Patient attended activity once in a month.

Supplementary Table 4

Medians and ranges of all cognitive subtests and QUALIDEM subscores for all time points of measurement differentiated for intervention and control group.

	Cognitive stimulation (*n* = 12)			Usual care (*n* = 6)	
	Pre-intervention Median (range)	Post-intervention Median (range)	Follow-up Median (range)	Pre-usual care Median (range)	Post-usual care Median (range)
Cognition					
CERAD semantic verbal fluency ("animals")[a]	8.00 (4 – 11)	8.50 (4 – 14)	5.00 (2 – 14)[3]	8.50 (3 – 14)	6.50 (4 – 11)
CERAD Boston Naming Test[a]	11.50 (5 – 14)	12.50 (9 – 14)	13.00 (8 – 13)[3]	9.50 (7 – 13)	10.00 (5 – 13)
CERAD MMSE total score[a]	17.50 (5 – 26)	15.50 (8 – 26)	20.00 (5 – 25)[3]	18.50 (11 – 25)	18.00 (5 – 26)
CERAD MMSE orientation[a]	5.00 (1 – 9)	4.50 (2 – 9)	7.00 (1 – 9)[3]	5.00 (4 – 9)	5.00 (1 – 9)
CERAD MMSE encoding[a]	3.00 (0 – 3)	2.00 (1 – 3)	2.00 (1 – 3)[3]	3.00 (2 – 3)	2.00 (0 – 3)
CERAD MMSE attention[a]	4.00 (0 – 5)	2.50 (0 – 4)	4.00 (0 – 5)[3]	3.50 (0 – 5)	3.50 (0 – 5)
CERAD MMSE memory[a]	1.00 (0 – 3)	1.00 (0 – 3)	0.00 (0 – 2)[3]	0.50 (0 – 2)	1.50 (0 – 3)
CERAD MMSE language[a]	6.00 (1 – 8)	6.00 (2 – 8)	6.00 (1 – 8)[3]	5.50 (3 – 8)	6.50 (1 – 8)
CERAD MMSE constructional praxis[a]	0.00 (0 – 1)	0.00 (0 – 1)	0.00 (0 – 0)[3]	0.00 (0 – 1)	0.00 (0 – 1)
CERAD word list learning[a]	7.00 (4 – 13)	9.00 (4 – 17)	9.00 (3 – 17)[3]	7.50 (4 – 11)	7.00 (6 – 12)
CERAD word list savings[a]	0.00 (0 – 200)	60.00 (0 – 100)[4]	75.00 (0 – 134)[3]	42.00 (0 – 100)	55.00 (0 – 200)
CERAD word list recall[a]	0.00 (0 – 4)	2.50 (0 – 6)	4.00 (0 – 6)[3]	1.00 (0 – 4)	2.00 (0 – 4)
CERAD word list intrusions[b]	3.00 (0 – 8)	2.00 (0 – 9)	2.00 (0 – 7)[3]	2.50 (2 – 8)	3.00 (0 – 8)
CERAD word list recognition discriminability[a]	65.00 (45 – 100)	72.50 (45 – 95)	85.00 (60 – 95)[3]	70.00 (45 – 90)	58.00 (45 – 100)
CERAD constructional praxis[a]	5.00 (0 – 9)	6.50 (2 – 11)	6.00 (0 – 11)[3]	6.50 (4 – 9)	4.00 (0 – 9)
CERAD constructional praxis recall[a]	1.00 (0 – 6)	0.00 (0 – 10)	1.00 (0 – 7)[2]	0.00 (0 – 3)	0.00 (0 – 6)
CERAD constructional praxis savings[a]	23.50 (0 – 100)	0.00 (0 – 111)	16.50 (0 – 117)[2]	0.00 (0 – 33)	0.00 (0 – 100)
Letter fluency test ("P-words")[a]	3.50 (2 – 15)	3.50 (1 – 13)	4.00 (1 – 10)[3]	6.50 (2 – 14)	5.00 (2 – 15)
Clock Drawing Test[a]	2.00 (1 – 5)	3.00 (1 – 9)	4.00 (1 – 5)[3]	3.00 (2 – 4)	2.00 (1 – 4)
Quality of life					
QUALIDEM A (care relationship)[a]	13.50 (5 – 21)	12.00 (4 – 21)[4]	14.00 (6 – 21)[3]	14.00 (8 – 16)	13.50 (8 – 16)
QUALIDEM B (positive affect)[a]	13.00 (6 – 18)	13.00 (6 – 18)[4]	15.00 (7 – 18)[3]	14.00 (8 – 18)	11.50 (6 – 18)
QUALIDEM C (negative affect)[a]	5.00 (2 – 9)	5.00 (2 – 9)[4]	5.00 (3 – 9)[3]	5.00 (4 – 9)	7.00 (4 – 9)
QUALIDEM D (restless tense behaviour)[a]	5.00 (4 – 9)[4]	5.00 (1 – 9)[4]	5.00 (2 – 9)[3]	5.00 (2 – 7)[1]	5.50 (4 – 9)
QUALIDEM E (positive self-image)[a]	5.00 (2 – 8)	6.00 (4 – 9)[4]	6.00 (3 – 8)[3]	5.00 (3 – 9)	4.50 (3 – 8)
QUALIDEM F (social relations)[a]	13.00 (3 – 17)	12.00 (8 – 18)[4]	12.00 (6 – 18)[3]	13.50 (9 – 16)	13.00 (11 – 16)
QUALIDEM G (social isolation)[a]	6.50 (3 – 9)	6.00 (5 – 9)[4]	6.50 (3 – 9)[3]	8.00 (6 – 9)[1]	6.50 (5 – 9)
QUALIDEM H (feeling at home)[a]	7.00 (3 – 11)[4]	10.00 (6 – 12)[4]	9.00 (4 – 12)[3]	6.00 (5 – 10)	8.00 (3 – 10)[1]
QUALIDEM I (have something to do)[a]	3.00 (0 – 5)	3.00 (0 – 4)[4]	2.00 (0 – 5)[3]	4.00 (0 – 6)	2.50 (0 – 5)

No results are presented for the TMT as only 4 patients successfully performed both subtests.

Abbreviations: CERAD, Consortium to Establish a Registry for Alzheimer's Disease; MMSE, Mini-Mental State Evaluation.

[a] Higher scores indicate a better performance.

[b] Lower scores indicate a better performance.

[1] *n* = 5

[2] *n* = 8

[3] *n* = 9

[4] *n* = 11

Supplementary material: references

[1] K.A. Welsh, N. Butters, R.C. Mohs, D. Beekly, S. Edland, G. Fillenbaum, A. Heyman, The Consortium to Establish a Registry for Alzheimer's Disease (CERAD). Part V. A normative study of the neuropsychological battery, Neurology 44(4) (1994) 609-614.

[2] C. Aebi, Validierung der neuropsychologischen Testbatterie CERAD-NP: eine Multi-Center Studie, University of Basel, Basel, 2002.

[3] F.O. Spreen, A.L. Benton, Manual of instructions for the Neurosensory Center Comprehensive Examination for Aphasia, University of Victoria, Victoria, British Columbia, 1977.

[4] R. Reitan, Trail-making test, Reitan Neuropsychology Laboratory, Arizona, 1979.

[5] E.H. Seo, D.Y. Lee, J.H. Lee, I.H. Choo, J.W. Kim, S.G. Kim, S.Y. Park, J.H. Shin, Y.J. Do, J.C. Yoon, J.H. Jhoo, K.W. Kim, J.I. Woo, Total scores of the CERAD neuropsychological assessment battery: validation for mild cognitive impairment and dementia patients with diverse etiologies, Am J Geriatr Psychiatry. 18(9) (2010) 801-809.

[6] K.I. Shulman, R. Shedletsky, I.L. Silver, The challenge of time: Clock-drawing and cognitive function in the elderly, Int J Geriatr Psychiatry 1 (1986) 135-140.

[7] S. Strotzka, Der Uhrentest, Z prakt Augenheilkd 35 (2014) 383-386.

[8] J.L. Cummings, The Neuropsychiatric Inventory: assessing psychopathology in dementia patients, Neurology 48(5 Suppl 6) (1997) S10-S16.

[9] J.I. Sheikh, J.A. Yesavage, Geriatric Depression Scale (GDS): recent evidence and development of a shorter version, in: T.L. Brink (Ed.), Clinical gerontology: a guide to assessment and intervention, Haworth Press, New York, 1986, pp. 165-173.

[10] G.S. Alexopoulos, R.C. Abrams, R.C. Young, C.A. Shamoian, Cornell Scale for Depression in Dementia, Biol Psychiatry 23(3) (1988) 271-284.

[11] F.I. Mahoney, D.W. Barthel, Functional Evaluation: The Barthel Index, Md State Med J 14 (1965) 61-65.

[12] R. de Haan, M. Limburg, J. Schuling, J. Broeshart, L. Jonkers, P. van Zuylen, [Clinimetric evaluation of the Barthel Index, a measure of limitations in dailly activities], Ned Tijdschr Geneeskd 137(18) (1993) 917-921.

[13] EuroQol Group, EuroQol--a new facility for the measurement of health-related quality of life, Health Policy 16(3) (1990) 199-208.

[14] T.P. Ettema, R.M. Droes, J. de Lange, G.J. Mellenbergh, M.W. Ribbe, QUALIDEM: development and evaluation of a dementia specific quality of life instrument. Scalability, reliability and internal structure, Int J Geriatr Psychiatry 22(6) (2007) 549-556.

[15] T.P. Ettema, R.M. Droes, J. de Lange, G.J. Mellenbergh, M.W. Ribbe, QUALIDEM: development and evaluation of a dementia specific quality of life instrument--validation, Int J Geriatr Psychiatry 22(5) (2007) 424-430.

[16] H. Verbeek, S.M. Zwakhalen, E. van Rossum, T. Ambergen, G.I. Kempen, J.P. Hamers, Dementia care redesigned: Effects of small-scale living facilities on residents, their family caregivers, and staff, J Am Med Dir Assoc 11(9) (2010) 662-670.
